# Ex Vivo Modulation of the BNST by Parabrachial CGRP Projections is Decreased After a History of Stress-Induced Anxiety

**DOI:** 10.1111/ejn.70268

**Published:** 2025-11

**Authors:** L. S. Kayat, N. Petersen, C. E. Van Doorn, B. P. Nabit, J. B. Tyree, S. W. Centanni, A. A. Jaramillo

**Affiliations:** 1 School of Medicine, Vanderbilt University, Nashville, Tennessee, USA; 2 Vanderbilt Brain Institute, Nashville, Tennessee, USA; 3 Vanderbilt Center for Addiction Research, Nashville, Tennessee, USA; 4 Department of Molecular Physiology and Biophysics, Vanderbilt University, Nashville, Tennessee, USA; 5 Department of Pharmacology, Vanderbilt University, Nashville, Tennessee, USA; 6 Department of Pharmaceutical Sciences, University of Kentucky College of Pharmacy, Lexington, Kentucky, USA; 7 College of Pharmacy, University of Kentucky, Lexington, Kentucky, USA

**Keywords:** abstinence, alcohol, anxiety, bed nucleus of the stria terminalis, parabrachial nucleus, stress

## Abstract

Activity in the parabrachial calcitonin gene-related peptide to bed nucleus of the stria terminalis (PBN[CGRP] → BNST) circuit associates with anxiety-like behavior. To test whether previous stress-induced anxiety is associated with neuronal adaptations in the PBN (CGRP) → BNST circuit, C57 mice were exposed to 4 days of daily forced swim stress (FSS). The novelty-suppressed feeding test (NSFT) demonstrated repeated FSS increased anxiety-like behavior. To assess if repeated FSS potentiates anxiety-like behavior in mice with dysregulated affect, mice received chronic intermittent ethanol vapor followed by FSS in abstinence. Anxiety-like behavior measured by NSFT was potentiated by repeated FSS in alcohol-abstinence. To measure neuroadaptations associated with a history of stress, *Calca*^*CRE*^ mice, with the Cre-dependent hM3D(Gq) DREADDs, in the PBN and the GFP-based Ca2+ sensor GCaMP7f in the bed nucleus of the stria terminalis (BNST), were exposed to repeated FSS and anxiety-like behavior measured with the NSFT. Weeks later, neurotransmission was measured by recording GCaMP7f fluorescence in ex vivo BNST slices. hM3D(Gq) DREADDs in PBN (CGRP) projections were activated via bath application of clozapine-n-oxide. PBN (CGRP) projections increased GCaMP7f spike frequency in the BNST in naïve conditions but not after a history of repeated FSS. Post-activation of PBN (CGRP) projections decreased GCaMP frequency and amplitude in the BNST, which was potentiated by a history of repeated FSS. These findings extend the anxiogenic role of PBN (CGRP) neurons by demonstrating neurotransmission in the PBN (CGRP) → BNST circuit is dysregulated after a stress-induced anxiogenic state. Given the potential of CGRP-related therapies, future studies will investigate the role of CGRP within the PBN → BNST circuit in inducing anxiety-related neuroadaptations.

## 1 | Introduction

Stress neurocircuits work in synchrony to respond to stimuli that are perceived as dangerous. The detection of a stressor and initiation of behavior are modulated in part by neurons expressing calcitonin gene-related peptide (CGRP) in the parabrachial nucleus (PBN [CGRP]; Palmiter 2018). In threat-associated or pain contexts, activating the PBN (CGRP) can induce an affective phenotype (Han et al. 2015; Campos et al. 2017; Campos et al. 2018; Jaramillo et al. 2020) driven primarily by projections to the extended amygdala (EA), bed nucleus of the stria terminalis (BNST), and central amygdala (CeA) (Han et al. 2015; Palmiter 2018; Bowen et al. 2020; Jaramillo et al. 2020, 2021). The PBN (CGRP) projections to the CeA predominantly modulate affective responses to noxious stimuli and conditioned hyperalgesia (Palmiter 2018; Neugebauer et al. 2020; Jaramillo et al. 2021), which are associated with neuroadaptations (Han et al. 2005; Shinohara et al. 2017; Neugebauer et al. 2020). The PBN (CGRP) projections to BNST modulate affective responses in stress-inducing contexts (Bowen et al. 2020; Jaramillo et al. 2021). However, it is unknown if neuroadaptations occur in PBN (CGRP) → BNST.

Activating the PBN (CGRP) → BNST circuit (Chen et al. 2018; Bowen et al. 2020) or CGRP infusions in the BNST (Sink et al. 2011; Sink et al. 2013) induce anxiety-like behavior and physiological responses (Bowen et al. 2020), whereas inhibition decreases anxiety-like behavior (Sink et al. 2011; Sink et al. 2013). The role of CGRP in the BNST extends to models for dysregulated negative affect (e.g., substance use disorders, SUD), as alcohol intake increased CGRP expression in anteroventral but decreased in anteromedial BNST (Rossetti et al. 2019). Inhibiting PBN projections in the BNST decreases excitatory postsynaptic current (EPSC) amplitude after a single stress episode (Fetterly et al. 2019), suggesting changes in PBN (CGRP) → BNST neurotransmission are sensitive to stress. It is unknown if CGRP-containing PBN projections affect synaptic activity in the BNST in stress models that exhibit heightened anxiety. The CGRP-containing PBN (EA) circuits are highly interconnected, as BNST-projecting PBN (CGRP) neurons collateralize to the CeA (Bowen et al. 2020; Zhang et al. 2025) and share fiber pathways (Dobolyi et al. 2005). In pain-inducing contexts, the PBN (CGRP) → CeA demonstrates changes in synaptic activity (Han et al. 2005; Shinohara et al. 2017) and molecular expression (Hwang et al. 1995; Chou et al. 2022; Allen et al. 2023). PBN (CGRP) → CeA is also affected in models for SUD, as alcohol preferring rats demonstrate increased CGRP peptide and receptor expression in CeA (Hwang et al. 1995). Due to the strong contribution of PBN (CGRP) on CeA and BNST in modulating affective behavior, it is likely that, similar to pain-induced neuroadaptations in the PBN (CGRP) → CeA (Neugebauer et al. 2020), anxiety-induced neuroadaptations occur in PBN (CGRP) → BNST. Given the limited number of studies on synaptic activity in the PBN (CGRP) → BNST circuit, this study will build on our mechanistic understanding of PBN (CGRP) projections in the EA by focusing on investigating anxiety-induced synaptic changes in the PBN (CGRP) → BNST circuit.

To understand how PBN (CGRP) projections drive synaptic activity after exposure to an anxiogenic state, we focus on the BNST, as in the absence of sustained pain, the BNST circuit plays a prominent role in anxiety-like behavior, relative to the CeA (Davis et al. 2010; Lebow and Chen 2016; Palmiter 2018; Jaramillo et al. 2021). We investigate if PBN (CGRP) → BNST neurotransmission is dysregulated after an anxiogenic state by using daily exposure to forced swim stress (FSS) and testing anxiety-like behavior with the novelty-suppressed feeding test (NSFT). Additionally, we test the effect of repeated FSS on NSFT in a model of alcohol-induced anxiogenic state in abstinence, as dysregulations in FSS and NSFT behavior associate with dysregulated BNST neurotransmission in negative affective states, such as alcohol abstinence (Vranjkovic et al. 2018; Centanni et al. 2019). Therefore, we use FSS and NSFT to induce an anxiogenic state. Postbehavior, we monitor global PBN (CGRP) → BNST neurotransmission in ex vivo BNST slices. In summary, these data demonstrate that a history of repeated FSS, in naïve conditions or alcohol abstinence, induces an anxiety-like phenotype. Additionally, weeks after a state of FSS-induced anxiety-like behavior, measuring ex vivo BNST activity demonstrated dysregulated BNST modulation by the PBN (CGRP), suggesting PBN (CGRP) → BNST is sensitive to neuroadaptations associated with a previous anxiogenic state.

## 2 | Materials and Methods

### 2.1 | Animals

Male and female C57BL/6J mice (Jackson Laboratories, Bar Harbor, ME, United States) were used for Experiment 1. Male C57BL/6J mice (Jackson Laboratories, Bar Harbor, ME, United States) were used for Experiment 2. Male *Calca*^CRE^ mice were used for Experiment 3. *Calca*^CRE^ mice are a heterozygous genetic knock-in mouse model that express Cre recombinase at the *Calca* locus with a C57BL/6 J background (see Carter et al. 2013 for *Calca*^CRE^ generation information). The exact genotype is Calca-WT (+) and Cre (+) with +/− genotype, indicating a half positive, heterozygous genotype. Mice were generously donated by Dr. Richard Palmiter and were bred in-house. All mice were approximately 10–12 weeks old at the start of experiments. Mice were single (Experiments 1 and 3) or group housed (Experiment 2) with water and food available ad lib in the home cage until the start of NSFT food restriction. The colony room was maintained on a 12-h light/dark cycle (lights on at 06:00) under controlled temperature (20°C–25°C) and humidity (30%–50%) levels. All behavioral experiments were conducted during the light phase. Ethanol exposure was conducted in the dark phase. Animals were under continuous care and monitoring by veterinary staff from the Vanderbilt Division of Animal Care or University of Kentucky Division of Lab Animal Resources. All procedures were carried out in accordance with the NIH Guide to Care and Use of Laboratory Animals and institutional guidelines and approved by the Institutional Animal Care and Use Committee at Vanderbilt University and the University of Kentucky.

### 2.2 | Behavioral Testing

#### 2.2.1 | Chronic Intermittent Ethanol (CIE)

As previously described (Silberman et al. 2013), mice received a daily intraperitoneal (IP) injection of pyrazole (1 mmol/kg) combined with 1.6 g/kg ethanol to inhibit alcohol metabolism and were placed in an ethanol vapor (95% ethanol at 5 L/min) enclosure (VIPER system, LJari, San Diego, CA, United States) for 16 h per day during the dark phase and then returned to standard animal housing for 8 h. A CIE cycle consisted of 4 days of ethanol vapor followed by a 3-day stay in regular animal housing. Air-exposed mice also received pyrazole, albeit without ethanol, as a control.

#### 2.2.2 | Forced Swim Stress (FSS)

During the light phase, mice were placed into a transparent cylindrical container (i.e., 5000-mL glass beaker) with water at room temperature (20.5°C–21.1°C) for 6 min. Water was changed in between animals. Swim sessions were recorded, albeit as noted in Experiment 2. Red overhead lighting was used with a white LED adjustable neck light aimed at the glass beaker. Lux was measured at the center of the surface of the water at 300–350 lx using a LX1010B Digital Light Meter (FTVOGUE, China). An observer blinded to treatment groups used Anymaze (Stoelting Co, Wood Dale, IL, United States) to analyze the last 4 min of the test for immobility.

#### 2.2.3 | Novelty-Suppressed Feeding Test (NSFT)

The 3-day NSFT was performed during the light phase, as previously described (Samuels and Hen 2011; Jaramillo et al. 2020). Briefly, mice were food restricted for 48 h in their home cage except for 2-h food access 22–24 h before testing. On test day (i.e., 48 h), mice were acclimated to the testing room for 1 h. The testing apparatus, 50 × 50 cm arena, contained fresh home cage bedding and a single food pellet at the center. Red overhead lighting was used with a white LED adjustable neck light aimed at the center of the arena. Lux was measured at the level of the bedding at the center of the arena at 300–450 lx with a LX1010B Digital Light Meter (FTVOGUE, China). Mice were placed in a corner of the arena. Mice were removed from the testing arena immediately after the first bite (i.e., consummatory food approach) and placed in their home cage with the food pellet from the test. The pellet was weighed after 10 min to confirm food consumption. Mice were returned to ad lib access to chow post testing. Latency to the first bite and food consumption were hand scored by an observer blinded to treatment groups. Tests were recorded with an overhead video camera. For a subset of mice, Anymaze (Stoelting Co, Wood Dale, IL, United States) was used to analyze additional measures in the behavioral videos (e.g., frequency and speed). Behavioral analyses were conducted by an observer blinded to treatment groups.

### 2.3 | Viral Injections/Stereotaxic Surgery

For Experiment 3, adult mice (> 8 weeks) were anesthetized with isoflurane (initial dose = 3%; maintenance dose = 1.5%), and surgery was performed using a stereotaxic frame with a heating pad to maintain mouse body temperature (Leica Biosystems, Nussloch, Germany). Coordinates from bregma were used to target the BNST (AP = 0.14, ML ± 0.88, DV = −4.18, 15.03° angle) and the PBN (AP = −5.34, ML ± 1.31, DV = −3.37, 15.03° angle). Care was taken to prevent eye drying by applying artificial tears ocular lubricant (Akorn, Lake Forest, IL, United States) and reapplying as needed throughout the surgery. For viral injections, 300 nL (40 nL/min) was infused, and the needle remained in place for an additional 5 min before withdrawal. The green fluorescent protein (GFP)–based calcium indicator AAV9-hSyn-Gcamp7f (GCaMP; lot v69477: 2.0 × 10^13^ GC/mL; lot v48294: 2.6 × 10^13^ GC/mL, lot v136635: 3.5 × 10^13^ GC/mL, Addgene, Watertown, MA, United States) was bilaterally infused in the BNST. The Cre-dependent, excitatory Designer Receptors Exclusively Activated by Designer Drugs (DREADDs) AAV5-hSyn-DIO-hM3D-mCherry (hM3D[Gq]; lot v30818: 7.8 × 10^12^ GC/mL; lot v112948: 2.5 × 10^13^ GC/mL, lot v172791: 2.6 × 10^13^ GC/mL, Addgene, Watertown, MA, United States) was bilaterally injected in the PBN. Mice were given 0.9% saline for fluid maintenance postoperatively, and body weights were tracked daily to ensure body weight was maintained. Mice were treated with Alloxate (2.5 mg/kg, S.C.) for 72 h after surgery and allowed to recover for at least 1 week before further experimentation.

### 2.4 | Tissue Preparation

As described (Pnevmatikakis et al. 2016; Zhou et al. 2018; Brown et al. 2023), mice in Experiment 3 were transcardially perfused with carboxygenated ice-cold cutting solution, consisting of (in mM): 92 N-Methyl-D-glucamine (NMDG), 2.5 KCl, 20 HEPES, 10 MgSO4·7H2O, 1.2 NaH2PO4, 0.5 CaCl2·2H2O, 25 glucose, 3 Na^+^-pyruvate, 5 Na^+^-ascorbate, 2 Thiourea, 30 NaHCO3, and 5 N-acetylcysteine. Coronal brain slices were obtained at 200-μm thickness using a vibrating Leica VT1000S microtome (Leica Biosystems, Nussloch, Germany). Brain slices recovered for 10–15 min in a 34°C chamber containing carboxygenated cutting solution and then transferred to a holding chamber with oxygenated artificial cerebrospinal fluid (aCSF) solution consisting of (in mM): 124 NaCl, 2.5 KCl, 2.5 CaCl2·2H2O, 5 HEPES, 2 MgSO4, 1.2 NaH2PO4, 12.5 glucose, 24 NaHCO3, and 0.4 L-ascorbic acid for 1 h at room temperature. After obtaining BNST-containing slices for ex vivo recordings, the remaining posterior portion of the brain was submerged in 4% paraformaldehyde in phosphate-buffered saline (PBS) for 24 h followed by 30% sucrose in phosphate-buffered saline (PBS) at 4°C. Coronal sections were cut on a cryostat (Thermo Scientific, Waltham, MA, United States, HM 525NX) in Optimal Cutting Temperature solution (VWR, Radnor, PA, United States) at a thickness of 40 μm and stored in PBS at 4°C.

### 2.5 | GCaMP Recordings

BNST-containing slices from Experiment 3 were placed in a perfusion chamber (Warner Instruments, Hamden, CT, United States) with carboxygenated aCSF (30°C), at a rate of 3 mL/min, and visualized using a 20X/0.5W water immersion objective (Olympus, Breinigsville, PA, United States). To emit blue light and record green fluorescence, optical excitation from a 470-nm LED (Thorlabs, Newton, NJ, United States, M470L3-C1) was driven by a T-Cube LED Driver (LEDD1B, Thorlabs, Newton, NJ, United States) and passed through two Neutral Density Filters, U-25ND25 and U-25ND50, (Olympus, Breinigsville, PA, United States) and an EN-GFP filter cube (Olympus, U-N410107, C26986). Slices were exposed to constant light at 75%–100% power for at least 10 min before recordings. Prior to recordings, the experimenter visually verified GCaMP using GFP autofluorescence and hM3D mCherry using mCherry autofluorescence in BNST slices. After recordings, BNST GCaMP expression was validated as described in immunohistochemistry methods (Figure 4a). hM3D projection expression in the PBN was verified with autofluorescence (Figure 4b). Only mice with accurate viral injections were included in the analysis and data presentation. Visual recordings were obtained using a Hamamatsu ORCA-Spark camera and HCimageLive software (Hamamatsu Photonics, Hamamatsu City, Shizuoka Pref., Japan). GCaMP measurements and recording parameters were consistent across all groups. High-speed streaming imaging parameters were set at a gain of 240, 200 ms exposure, binning of 2 × 2, and a rate of 5 frames per second (fps). Recordings lasted a total of 15 min, consisting of 5-min aCSF (Baseline), 5 min of clozapine-n-oxide (CNO) (10 μM; CNO wash-on), and 5 min of aCSF (CNO wash-off) for a total of 4500 frames. Recordings were conducted by an experimenter blinded to treatment groups.

### 2.6 | GCaMP Analysis

GCaMP recordings were analyzed as previously described in Brown et al. (2023). Briefly, to preprocess the videos for GCaMP analysis in Experiment 3, ImageJ was used to combine individual TIFF images and spatially downsample the frames. The nonrigid motion correction of calcium imaging data (NoRMCorre) algorithm was used in MATLAB for motion correction. Open-source code for NoRMCorre is freely accessible (https://github.com/flatironinstitute/NoRMCorre/tree/master) and described in Pnevmatikakis et al. (2016). Next, data were extracted using automated ROI detection with signal deconvolution, denoising, and demixing using constrained nonnegative matrix factorization for microendoscopic data (CNMF-E) analysis (Zhou et al. 2018) in MATLAB (MathWorks, Natick, MA, United States). Open-source code for CNMF-E is freely accessible from (https://github.com/zhoupc/CNMF_E) and described in Zhou et al. (2018). CNMF-E analysis allows for *Z*-score calculation and detection of calcium events in single cells. A *Z* score of > 5 determined an event (i.e., spike). The change in spikes per min (frequency) of each cell was calculated by subtracting the instantaneous spikes/min from the average spikes per minute during baseline (Minutes 1–5). A threshold of 20% was selected to determine cells that responded when the drug was on, allowing for grouping analysis based on increases or decreases in cell responses. Data processing was conducted by an observer blinded to treatment groups.

### 2.7 | Tissue Preparation for Viral Vector Confirmation

Following ex vivo recordings, BNST slices were processed with the Brain Blocking of Lipids and Aldehyde Quenching (BLAQ) procedure as described previously (Kupferschmidt et al. 2015). Sections were washed for 1 h in PBST (0.2% Triton X-100 in PBS), rinsed twice for 1 min in diH2O, quenched twice for 10 min in freshly prepared sodium borohydride (NaBH4; 5 mg/mL = 132 mm in diH2O; Sigma-Aldrich, St. Louis, MO, United States, #213462, 99%), rinsed again in diH2O (2 × for 1 min), incubated twice for 15 min in Sudan Black B solution (0.2% in 70% ethanol; Sigma-Aldrich, St. Louis, MO, United States, 199664), washed twice for 30 min in PBS, and incubated for 4 h in 10% normal donkey serum (NDS) (Jackson ImmunoResearch Laboratories, West Grove, PA, United States, 017–000–121) in PBST. Slices were incubated in primary antibody (1:1000 goat anti-GFP [Abcam, Waltham, MA, United States, ab6673] and mouse anti-mCherry [Abcam, Waltham, MA, United States, ab125096]) for 72 h at 4°C, washed four times in PBST for a total of 24 h at 4°C, and then incubated in secondary antibody (1:400, donkey antigoat [Jackson ImmunoResearch, West Grove, PA, United States, Alexa Fluor 488] and donkey antimouse [Jackson ImmunoResearch, West Grove, PA, United States, Alexa Fluor 555]) for 48 h at 4°C. Finally, slices were washed four times in PBST for a total of 24 h at 4°C, washed in PBS for 1 h at room temperature (RT), and then mounted on Fisher Plus slides (Fisher Scientific, Waltham, MA, United States) and cover slipped with Poly AquaMount (Polysciences, Warrington, PA, United States) when dry. GFP and mCherry immunofluorescence in the BNST were visualized, and images were obtained with a Zeiss LSM 880 confocal microscope using a 10X Apochromat Oil objective lens (Carl Zeiss AG, Oberkochen, Germany) (Figure 4a). PBN slices obtained post ex vivo tissue preparation were mounted on Fisher Plus slides and cover slipped with Poly AquaMount. mCherry autofluorescence was visualized, and images were obtained with an ECHO Revolve microscope (ECHO, San Diego, CA, United States) using 10X Apochromat (Figure 4b). All images were obtained and data processed by an observer blinded to treatment groups. Only mice with accurate viral injections were included in the GCaMP analysis.

### 2.8 | Experimental Procedures

#### 2.8.1 | Experiment 1: Examination of Repeated FSS on NSFT

**FIGURE 1 |:**
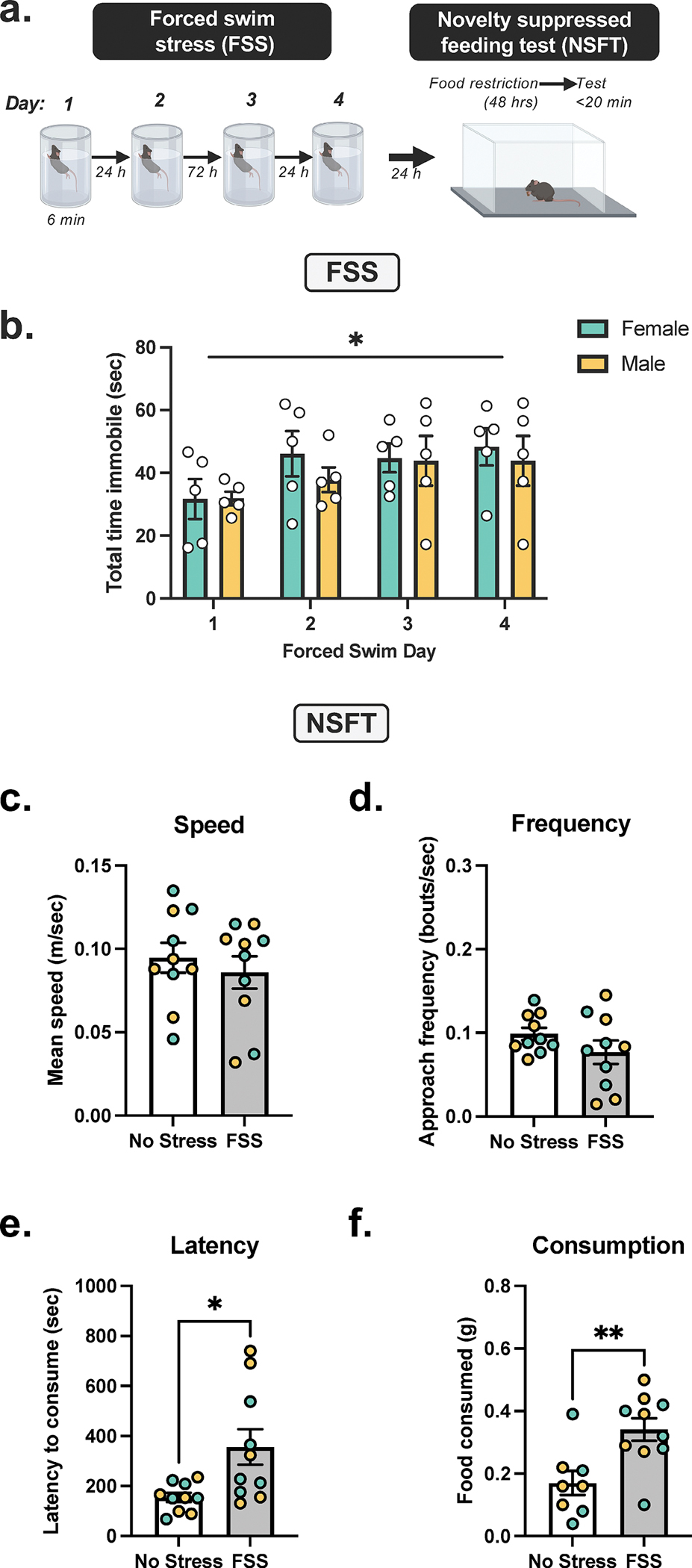
Repeated FSS increases latency to feed in NSFT and food consumption. (A) Experimental design demonstrating C57BL/6 J male and female mice, depicted by yellow and green data points, respectively, were exposed to 6-min daily forced swim stress (FSS) for 4 days followed by the novelty-suppressed feeding test (NSFT). (B) Total immobility was increased across 4 days of FSS in males and females, as demonstrated by a main effect of day. (C) Speed was decreased, and (D) frequency was unaffected by FSS. (E) Latency for consummatory approach and (F) post-NSFT food consumption were increased by FSS. Values on graphs represent mean ± SEM Two-way ANOVA (Tukey), *t* test (*n* = 10, *p = 0.01, **p = 0.003).

C57BL/6J male and female mice (*n* = 10) underwent a repeated FSS paradigm (Figure 1) as follows: one daily FSS session for 2 days, a 2-day incubation period (i.e., no FSS), and one daily FSS session for 2 days. Control mice (5 female and 5 males) remained undisturbed. Next, to measure anxiety-like behavior, NSFT was conducted.

#### 2.8.2 | Experiment 2: Examination of FSS on NSFT in Prolonged CIE Abstinence

**FIGURE 2 |:**
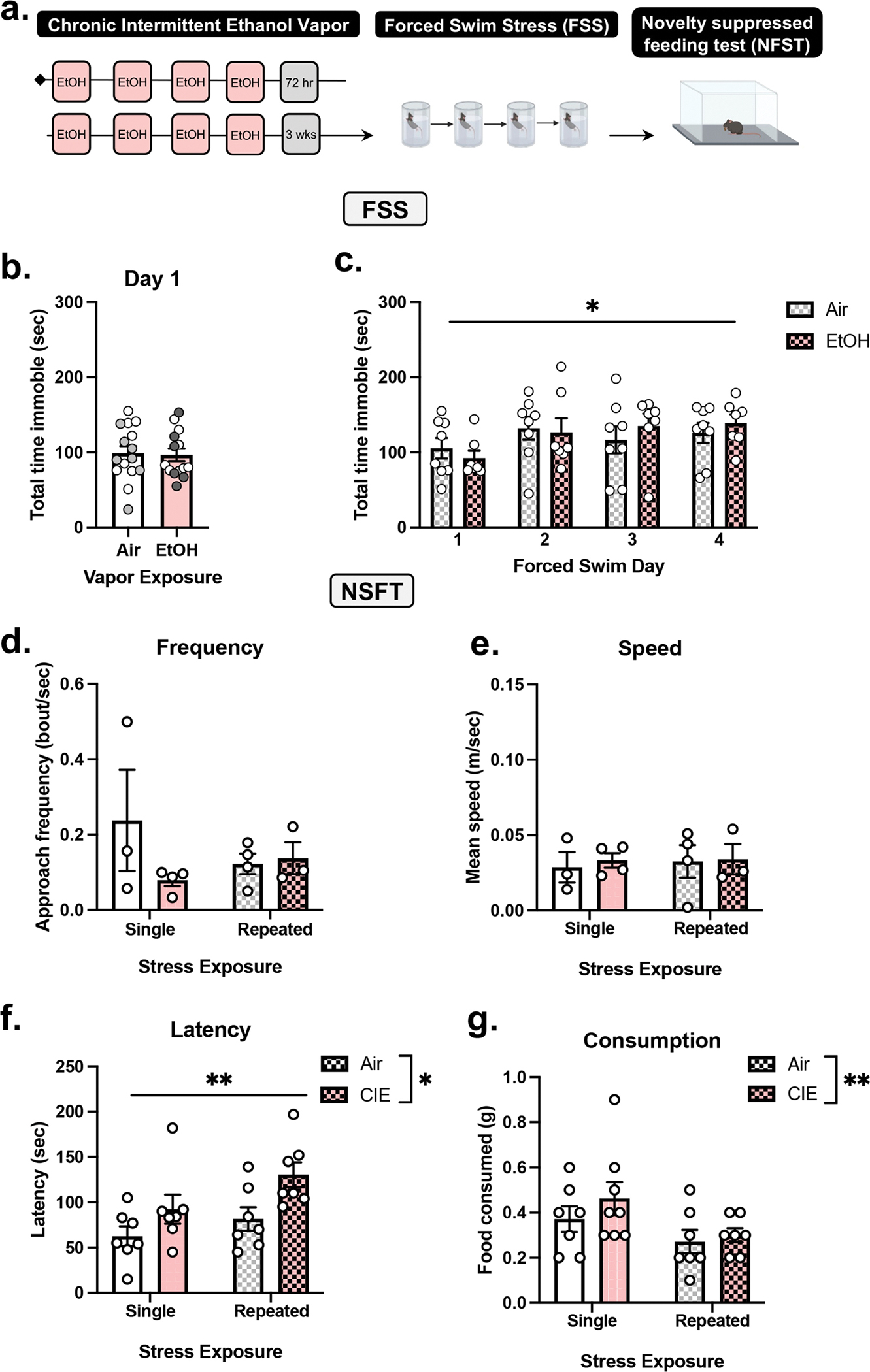
Repeated FSS in CIE abstinence increases latency to feed in NSFT. (A) Experimental design demonstrating C57BL/6J male mice was exposed to chronic intermittent ethanol (CIE) vapor exposure (one cycle = 4 days of 16 h on followed by 4 h of withdrawal) or air exposure (controls). (B) At 3 weeks of abstinence from air or CIE vapor exposure, all mice were exposed to 6 min forced swim stress (FSS) for 4 days followed by novelty-suppressed feeding test (NSFT). Next, mice were divided into repeated FSS (gray) and single FSS groups (white). (C) A history of CIE did not affect total time immobile in the first forced swim exposure relative to air exposed controls. Across FSS total time immobile was increased in Air and CIE-exposed groups demonstrated by a main effect of day. (D) In NSFT, speed and frequency were unaffected by CIE or repeated FSS, as analyzed in a subset of mice. (F) Repeated FSS and CIE increased latency for consummatory approach in NSFT relative to controls (i.e., no stress and air, respectively). (G) CIE increased food consumed post-NSFT relative to air control demonstrated by a main effect of vapor exposure. Values on graphs represent mean ± SEM. Two-way ANOVA (Tukey), *t* test (*p = 0.05, **p < 0.008).

Two cohorts of C57BL/6J male mice (total *n* = 29) underwent CIE or Air (controls) vapor exposure (Figure 2a). Next, in the third week of abstinence, mice in the repeated FSS group underwent repeated FSS as follows (i.e., 4 days of FSS): one daily FSS session for 2 days, a 2-day incubation period (i.e., no FSS), and one daily FSS session for 2 days. The nonrepeated FSS group (controls) underwent 1 day of FSS (denoted by white in Figure 2b) and remained undisturbed for the remainder of the experiment. Next, all mice underwent the 3-day NSFT paradigm. NSFT was video recorded in a subset of mice (*n* = 14), allowing for detailed analysis (i.e., frequency, speed, and immobility).

#### 2.8.3 | Experiment 3: Investigation of FSS on NSFT and BNST Activity

**FIGURE 3 |:**
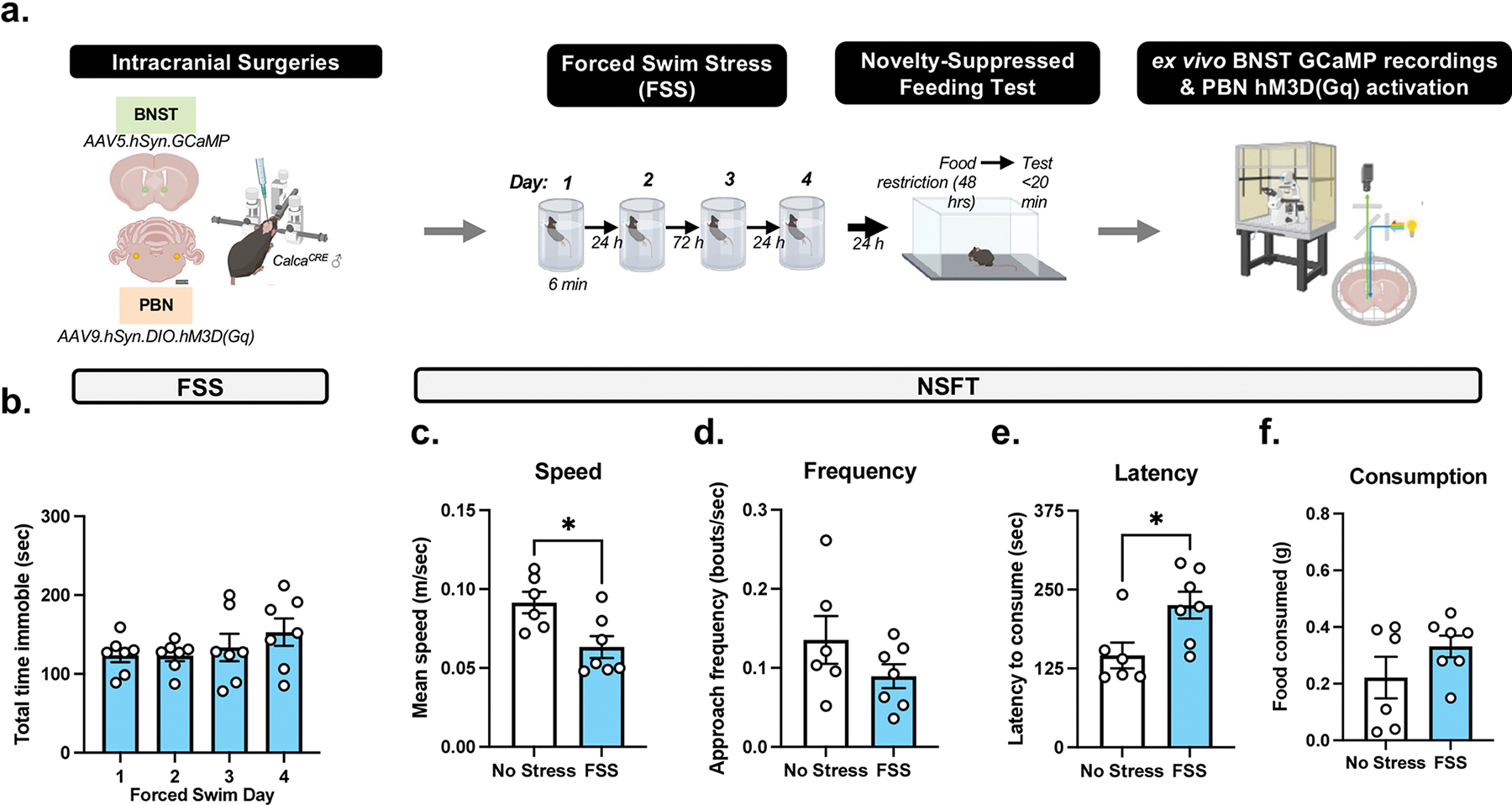
Repeated FSS increases latency to feed in NSFT prior to ex vivo BNST recordings. (A) Experimental design demonstrating *Calca*^CRE^ male mice receive bilateral infusions of the Ca2+ sensor GCaMP in the BNST, Cre-dependent excitatory hM3D(Gq) in the PBN, and exposure to repeated forced swim stress (FSS) and novelty-suppressed feeding test (NSFT). Next, GCaMP fluorescence is recorded in ex vivo BNST-containing slice. (B) Total immobility increases across 4 days of FSS. (C) In NSFT, speed increased, and (D) frequency was unaffected by FSS. (E) FSS increases latency for consummatory approach in NSFT and (F) no effect on food consumed post-NSFT. Values on graphs represent mean ± SEM, *t* test (*p < 0.05).

**FIGURE 4 |:**
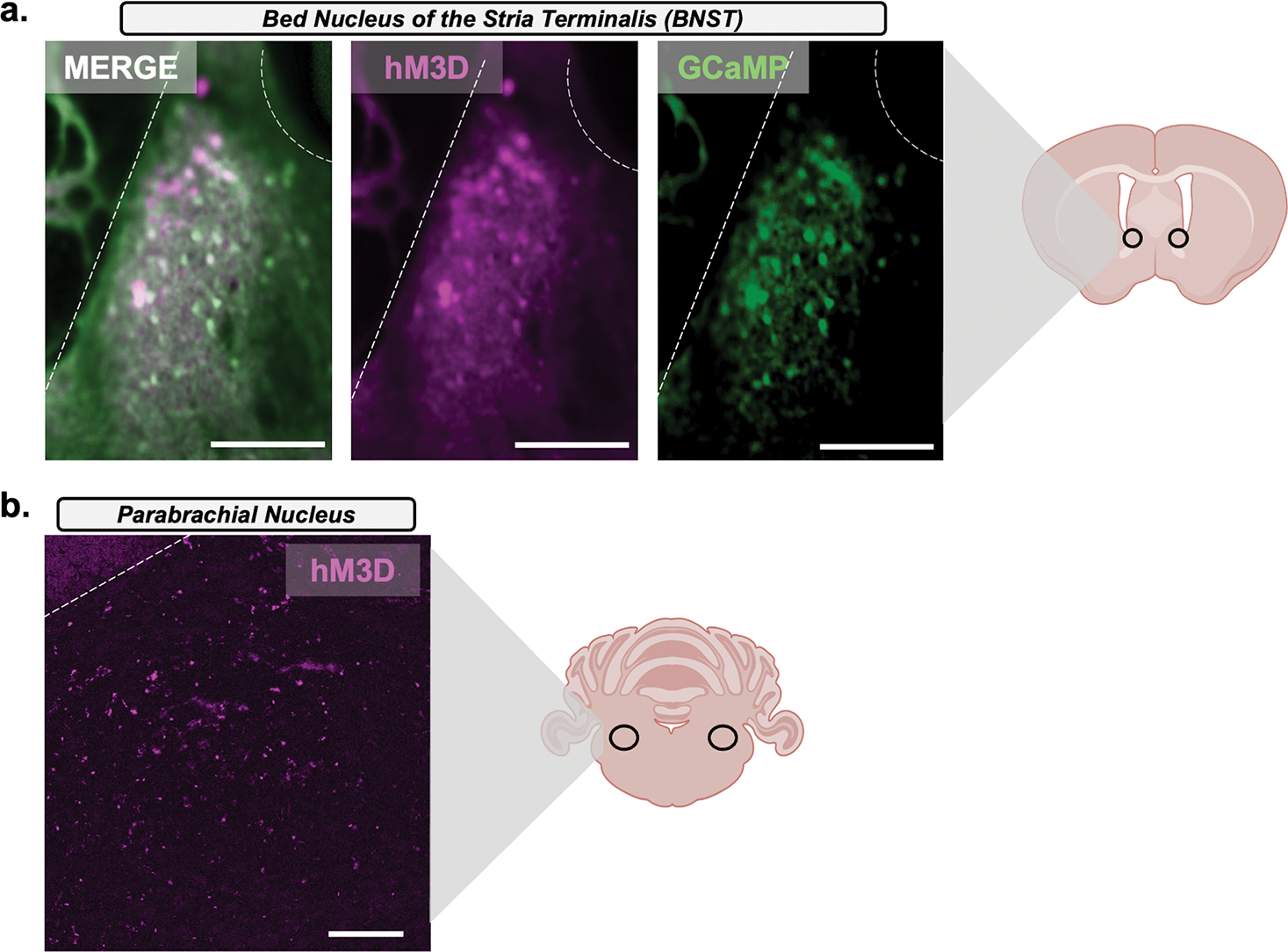
BNST GCaMP and PBN hM3D DREADD expression. After ex vivo recordings GCaMP expression and DREADD expression were verified. (A) Representative image showing the expression of GCaMP-eGFP (green) and hM3D-mCherry (purple) in the BNST in a post hoc fixed 200-μm-thick slice (200 μm scale). (B) Representative image showing expression of hM3D-mCherry autofluorescence in the PBN in a post hoc fixed 40-μm-thick slice (180-μm scale).

*Calca*^*CRE*^ male mice (*n* = 13) received bilateral injections of Cre-dependent hM3D DREADD in the PBN and GCaMP in the BNST (Figure 3a and Figure 4). Following recovery from surgery, mice underwent FSS as follows: one daily FSS session for 2 days, 2-day incubation period (i.e., no FSS), and one daily FSS session for 2 days. Next, the NSFT paradigm was conducted, and after 2–4 weeks, brain slices were extracted for ex vivo GCaMP recordings. Animals with errors due to GCaMP recordings (*n* = 3) or incorrect viral placement (*n* = 2) were omitted from GCaMP analysis.

### 2.9 | Drugs

CNO was diluted in aCSF (10 μM) and bath applied onto BNST-containing slices for ex vivo Ca2+ recordings in Experiment 3. Pyrazole + Ethanol (1 mmol/kg + 1.6 g/kg Ethanol, IP) or Pyrazole (1 mmol/kg, IP) was diluted in sterile saline prior to each exposure.

### 2.10 | Statistical Analysis

Data are represented as mean ± SEM, and all statistics were run using Prism 7 (GraphPad, Boston, MA, United States). Differences between groups were assessed using *t* tests, one-way analysis of variance (ANOVAs), and two-way ANOVAs. Repeated measures (RM) analysis and post hoc tests were conducted when appropriate. Significance was set at *p* ≦ 0.05.

## 3 | Results

### 3.1 | Experiment 1: FSS Increases Consummatory Approach in NSFT

We investigate FSS in C57BL/6J males and females. Comparison of total FSS session immobility relative to no stress controls demonstrates increased immobility across 4 days of exposure (Figure 1a), as two-way RM ANOVA demonstrates a significant main effect of day (*F*[3, 24] = 2.975, *p* = 0.0517). There was no main effect of sex (*F*[1, 8] = 0.345, *p* = 0.5734) and no sex by day interaction (*F*[3, 24] = 0.2690, *p* = 0.8471) on total immobility (Figure 1b). Two-way ANOVA of all NSFT data demonstrated no main effect of sex. Therefore, female and male data were combined for all further analysis as denoted by green and yellow symbols, respectively. Next, to investigate the effect of FSS, an unpaired *t* test analysis of NSFT behavior was performed. FSS significantly increased total distance traveled in NSFT relative to no stress controls (*t*_18_ = 2.841, *p* = 0.0108), albeit at a higher rate in females (*t*_8_ = 2.496, *p* = 0.0372 vs. *t*_8_ = 7.477, *p* < 0.0001). FSS did not affect approach frequency (Figure 1d; *t*_18_ = 1.393, *p* = 0.1807) and mean speed (*t*_18_ = 0.6666, *p* = 0.5135). Latency to consume was increased in FSS exposed mice relative to no stress controls (Figure 1e; *t*_18_ = 2.750, *p* = 0.0132). Posttest analysis of eight animals demonstrates FSS increased post-test food consumption relative to no stress controls (Figure 1f; *t*_16_ = 3.227, *p* = 0.0026).

### 3.2 | Experiment 2: Repeated FSS Potentiates NSFT Consummatory Approach Behavior in Prolonged Alcohol-Abstinence

To assess repeated FSS behavior in prolonged abstinence from CIE, C57BL/6J male mice underwent two cycles of CIE or Air (controls) vapor, single FSS or repeated FSS, and were tested on NSFT (Figure 2a). At 3 weeks of abstinence, forced swim immobility was measured. An unpaired *t* test analysis on total time immobile (*t*_27_ = 0.1765, *p* = 0.8612) revealed no significant effect of CIE vapor exposure compared to air exposed controls (Figure 2b). Next, groups were divided into single FSS (denoted in white) and repeated FSS groups (denoted in gray). The repeated FSS group underwent an additional 3 days of FSS (i.e., a total of 4 FSS). The single FSS group did not receive any additional FSS for the remainder of the experiment (i.e., a total of 1 FSS). Two-way RM ANOVA revealed a significant main effect of repeated FSS exposure (*F*[3, 39] = 4.876, *p* = 0.0057), as time immobile increased across FSS sessions (Figure 2c). There was no significant main effect of vapor exposure (*F*[1, 13] = 0.03577, *p* = 0.8529) nor vapor x day interaction (*F*[3, 39] = 1.163, *p* = 0.336).

Next, we investigate the effect of repeated FSS on NSFT behavior in CIE relative to air exposed mice. Two-way RM ANOVA of video recordings in a subset of mice (*n* = 3–4/group) shows there was no effect of repeated FSS and CIE vapor on average speed (Figure 2e; FSS: *F*[1, 10] = 0.1092, *p* = 0.7479; vapor: *F*[1, 10] = 0.06200, *p* = 0.8084; interaction: *F*[1, 10] = 0.02806, *p* = 0.8703) and approach frequency (Figure 2d; FSS: *F*[1, 10] = 1.354, *p* = 0.2716; vapor: *F*[1, 10] = 0.2176, *p* = 0.6508; interaction: *F*[1, 10] = 1.973, *p* = 0.1905). Two-way ANOVA of hand-scored data in all animals demonstrates CIE and repeated FSS increased latency to consume (Figure 2f), relative to Air and single FSS groups, as there was a main effect of repeated FSS (*F*[1, 24] = 8.449, *p* = 0.0077), vapor (*F*[1, 24] = 4.436, *p* = 0.0458), and no significant interaction (*F*[1, 24] = 0.4831, *p* = 0.4937). One animal was a significant outlier (*p* < 0.05, *z* = 2.41) and was omitted. Food consumed post-test was increased following CIE vapor (Figure 2g) as two-way ANOVA revealed a significant main effect of vapor (*F*[1, 24] = 6.154, *p* = 0.0205). There was no significant effect of FSS (*F*[1, 24] = 1.538, *p* = 0.2268) nor interaction (*F*[1, 24] = 0.5538, *p* = 0.4640).

### 3.3 | Experiment 3: FSS Increases NSFT Consummatory Approach Behavior and Potentiates PBN-Induced Decrease in BNST GCaMP Activity

To investigate FSS-induced changes in PBN (CGRP) → BNST activity, *Calca*^*CRE*^ male mice received bilateral injections of Cre-dependent hM3D DREADDs in the PBN and GCaMP in the BNST (Figures 3a and 4). One-way ANOVA of total time immobile demonstrates repeated FSS did not change immobility as there was no significant main effect of time (*F*[3, 18] = 1.249, *p* = 0.3215; Figure 3b). An unpaired *t* test demonstrated FSS had a significant effect on average speed (*t*_11_ = 2.869, *p* = 0.0153; Figure 3c) and no effect on approach frequency (*t*_11_ = 1.422, *p* = 0.1827; Figure 3d) in NSFT. An unpaired *t* test demonstrated a significant effect of FSS, as there was a significant increase in latency to consume (*t*_11_ = 2.666, *p* = 0.0220; Figure 3e) relative to no stress. There was no effect on food consumed (*t*_11_ = 1.385, *p* = 0.1935; Figure 3f) relative to no stress controls.

**FIGURE 5 |:**
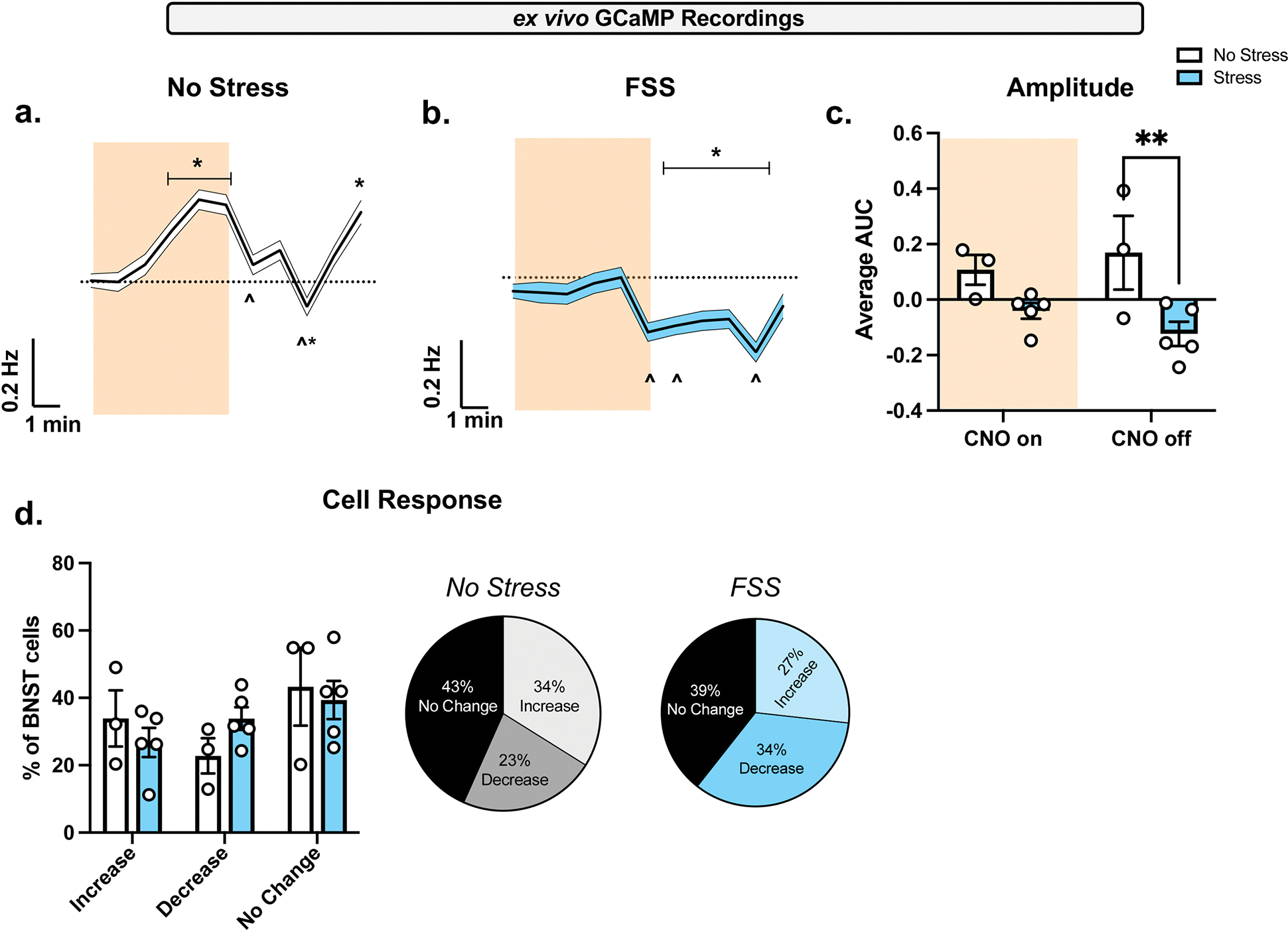
Increase in global BNST GCaMP frequency ex vivo induced by chemogenetic PBN activation is decreased with a history of FSS. After a repeated FSS and NSFT, GCaMP was measured in ex vivo BNST slices. Data represent averages per animal. (A) In no stress, bath applying hM3D ligand CNO 10 μM resulted in a main effect of CNO in calculated changes in GCaMP spike frequency. CNO wash-on increased frequency relative to baseline (*). CNO wash-off decreased frequency relative to CNO wash-on (^). At the end of CNO wash-off frequency increased relative to baseline (*) and CNO wash-on (^). (B) In FSS conditions, CNO wash-off decreased GCaMP spike frequency relative to baseline (*) and CNO wash-on (^), as demonstrated by a main effect of CNO. (C) CNO wash-off decreases AUC in FSS conditions, demonstrated by a main effect of stress exposure. (D) Averaged per animal, pie charts show individual cells were characterized by > 20% change in GCaMP fluorescence as an increase, decrease, or no response to CNO. One-way ANOVA (mean ± SD; Sidak; *p* < 0.05), or two-way ANOVA (mean ± SEM; Tukey; *p* < 0.03). All measurements were consistent across groups; baseline (0–4 min), CNO wash-on (5–10), and CNO wash-off (11–15).

Next, the effect of a history of FSS on PBN (CGRP) → BNST activity was investigated with GCaMP imaging in BNST-containing brain slices (Figure 5). GCaMP activity was recorded across all individual neurons and the ligand for hM3D, CNO 10 μM, was bath applied (orange shading). All measurements were consistent across groups: baseline (0–4 min), CNO (5–10), and CNO wash off (11–15). Relative to baseline, CNO increased the average global change in GCaMP spikes per min in the no stress group (Figure 5a), as one-way RM ANOVA demonstrates a significant main effect of CNO (*F*[14, 30,075] = 12.54, *p* < 0.0001). Sidak’s post hoc analysis demonstrates frequency increased with CNO (8–9 min) relative to baseline (*p* < 0.0240). At the start of CNO wash off (11, 13 min), frequency decreased relative to CNO (*p* < 0.0001). CNO wash off (15 min) increased frequency relative to baseline (*p* < 0.0001) and CNO (*p* < 0.0001). In the FSS group (Figure 5b), CNO decreased the average global change in GCaMP spikes per min relative to baseline, as one-way RM ANOVA (*F*[14, 26,580] = 11.21, *p* < 0.0001) demonstrates a significant main effect of CNO. Relative to baseline, Sidak’s post hoc analysis demonstrates frequency decreased with CNO wash off (11–15 min; *p* < 0.0458). Relative to CNO, frequency decreased with CNO wash off (10, 11, 14 min; *p* < 0.0304). A two-way RM ANOVA of area under the curve (AUC) in the FSS versus no stress group during CNO and CNO wash off (Figure 5c) demonstrates a significant main effect of FSS exposure (*F*[1, 6] = 8.236, *p* = 0.0284). Specifically, AUC was decreased in the FSS group relative to the no stress group during CNO wash off (*p* = 0.0064). We categorize BNST neurons based on > 20% change in GCaMP fluorescence. When looking across all individual recorded neurons in no stress and FSS groups (Figure 5d), we show varied responses to CNO with neurons exhibiting an increase, decrease, and no change.

## 4 | Discussion

The present findings demonstrate that the modulation of BNST activity by PBN (CGRP) projections is dysregulated weeks after a stress-induced anxiogenic state. Using repeated exposure to FSS, we delayed the initiation of consummatory approach in the conflict-inducing NSFT context. Postbehavior, the PBN (CGRP)-induced increase in ex vivo BNST Ca2+ spike frequency was blunted in mice previously exposed to repeated FSS. Additionally, ex vivo BNST Ca2+ spike frequency and amplitude were decreased during recovery from PBN (CGRP) activation in stress-naïve conditions. The decrease in BNST ex vivo Ca2+ activity was potentiated in mice with a history of FSS. Thereby demonstrating that neuronal activity in the BNST induced by PBN (CGRP) projections is dysregulated after exposure to an anxiogenic state.

We demonstrate repeated stress induces anxiety-like behavior in naïve conditions and in alcohol abstinence. Using the repeated stress exposure paradigm, we investigated if PBN (CGRP) → BNST synaptic activity changes after exposure to stress-induced anxiety-like behavior. First, to establish the circuit activity within PBN (CGRP) → BNST, via ex vivo Ca2+ recordings, we demonstrate selectively activating PBN (CGRP) projections increases global spike frequency in the BNST. Previous studies demonstrate exogenous CGRP administration increases stimulation-evoked BNST inhibitory postsynaptic current (IPSCs) amplitude (Gungor and Pare 2014). Optogenetic activation of global PBN projections (not specific to CGRP) induces EPSCs (Flavin et al. 2014), and chemogenetic inactivation decreases EPSC amplitude (Fetterly et al. 2019). By isolating CGRP-specific PBN projections, we demonstrate that circuit-specific ex vivo activation of PBN (CGRP) projections has a global excitatory effect on BNST frequency. Relative to previous literature, these findings suggest that activating the PBN (CGRP) → BNST circuit modulates single whole-cell activity that results in an increase in global BNST activity. The excitatory effect of PBN (CGRP) on global BNST activity is in line with in vivo studies (Jaramillo et al. 2020). Additional neuron-specific analysis demonstrates excitatory- and inhibitory-responding neurons encompass the global PBN (CGRP)-induced changes in BNST activity. Similarly, whole-cell ex vivo recordings demonstrate activation of PBN projections (not specific to CGRP) activates and inhibits BNST neurons (Flavin et al. 2014). Interestingly, BNST frequency changed post recovery from PBN (CGRP) activation, suggesting that the effects of PBN (CGRP) activation on BNST remain after PBN (CGRP) → BNST activation. One study demonstrates that modulating the PBN → BNST circuit has long-lasting effects after CNO activation (Fetterly et al. 2019). Given that PBN-induced inhibitory responding in the BNST is a result of feed-forward inhibition (Flavin et al. 2014), it is possible that the residual effects after PBN (CGRP) activation are mediated by the inhibitory-responding population. Due to the time constraints of the experiment, it is unknown when BNST activity returns to baseline. A population of BNST neurons did not respond after activating PBN (CGRP) projections, suggesting a subset of BNST neurons is not recruited by PBN (CGRP) projections. Similarly, activating PBN projections in the ex vivo BNST does not induce activity in all BNST cells (Fetterly et al. 2019). Together, the data provide a global view of the PBN (CGRP) → BNST circuit by demonstrating PBN (CGRP) has a global increase in neuron frequency that is accompanied by residual effects through modulation of excitatory- and inhibitory-responding BNST neurons.

To investigate if PBN (CGRP) → BNST circuit activity changes after a stress-induced anxiogenic state, the circuit was undisturbed prior to ex vivo analysis. In this manner, changes in PBN (CGRP) → BNST activity were associated with a history of repeated stress and not a history of circuit manipulation. Thereby, we demonstrate that, unlike stress-naïve conditions, activating PBN (CGRP) after a history of stress does not change global BNST frequency. However, like stress-naïve conditions, global BNST frequency decreased post-PBN (CGRP) activation, albeit to negative levels. Interestingly, global BNST amplitude was decreased with a history of repeated stress relative to stress-naïve conditions, suggesting a potentiated residual effect of PBN (CGRP) activation. Inhibiting PBN projections in the BNST decreases protein c-Fos (cFOS) in corticotropin-releasing factor (CRF) neurons after restraint stress, suggesting PBN → BNST circuit activity is sensitive after a stress episode (Fetterly et al. 2019). The role of CRF in modulating anxiety-like behavior (Maita et al. 2022) is in part through CGRP in the BNST (Sink et al. 2013). Studies that are not specific to CGRP demonstrate modulating PBN projections in the BNST occurs in part through CRF+ neurons (Fetterly et al. 2019). These studies and the dense colocalization of CRF+ neurons and CGRP terminals (Kozicz and Arimura 2001) suggest dysregulated activity in PBN (CGRP) → BNST may be associated with CRF+ neurons. These data demonstrate neuroadaptations in PBN (CGRP) → BNST associate with a history of a stress-induced anxiogenic state. Thereby, these data contribute to the PBN (CGRP) → EA literature by suggesting PBN (CGRP) → EA circuits are sensitive to an affective disturbance in the absence of noxious stimuli.

Our initial study demonstrates that repeated stress increased anxiety-like behavior and hyperphagia. Interestingly, our study investigating ex vivo PBN (CGRP) → BNST activity in surgerized subjects showed a history of repeated stress did not induce hyperphagia. Similarly, in ethanol and air exposed mice, repeated stress did not affect food consumption. Therefore, our model of stress-induced anxiety-like behavior demonstrates variable effects on food consumption that may be due to viral surgery or are occluded by alcohol exposure. In contrast, repeated FSS consistently induces an anxiogenic phenotype. We demonstrate repeated FSS exacerbated the anxiogenic state induced by alcohol abstinence. It is well established that negative affective states induced by alcohol abstinence associate with dysregulated activity in the BNST (Avery et al. 2016; Ch’ng et al. 2018; Vranjkovic et al. 2018; Centanni et al. 2019). It is unknown if PBN (CGRP) projections contribute to dysregulated BNST activity in alcohol abstinence. Alcohol decreases firing rate in PBN neurons (Liu et al. 2023), and alcohol intake induces changes in CGRP expression in the BNST (Rossetti et al. 2019), thereby suggesting PBN (CGRP) → BNST activity may be sensitive to alcohol exposure. Our findings demonstrating repeated stress increases anxiety-like behavior in abstinence that associates with dysregulated PBN (CGRP) → BNST activity, demonstrate the need for future studies to investigate if stress-induced dysregulations in PBN (CGRP) → BNST are potentiated in alcohol-abstinence.

The present study used DREADDs to activate PBN (CGRP) projections and GCaMP to measure activity. Unequal distribution of genetically modified tools is a caveat of techniques that rely on viral expression (e.g., DREADDs, GCaMP, and optogenetics). Thus, although our findings demonstrate DREADD expression was primarily colocalized with GCaMP neurons, the lack of response in the non-responding neurons may be due to their proximity to DREADD-expressing PBN (CGRP) projections. However, based on previous findings demonstrating PBN does not induce activity in all BNST cells (Fetterly et al. 2019), it is likely that non-responding BNST cells reflect that PBN (CGRP) only modulates a subset of neurons. An additional caveat of using DREADDs is the possibility that neurons that responded may have been recruited by off-target effects of CNO (i.e., not through DREADD activation). Our previous work using DREADDs and the *Calca*^*CRE*^ transgenic line to target PBN (CGRP) neurons demonstrates no behavioral effects of the DREADD ligand CNO in non-DREADD-expressing mice (e.g., mCherry controls; Jaramillo et al. 2020). However, the present study administered CNO directly to the tissue and not in behaving mice; thus, there is still a possibility that nanomolar concentration can have off-target effects (Jendryka et al. 2019). Future studies that do not depend on viral expression or CNO will provide more insight into the non-responding BNST population and confirm the effects of manipulating PBN (CGRP) neurons.

## 5 | Conclusions

Here, we confirm that PBN (CGRP) increases BNST activity through excitatory and inhibitory responding neurons. Moreover, post-PBN (CGRP) activation, BNST activity is decreased. We expand these findings in a stress-induced model of anxiety to demonstrate the excitatory effect of PBN (CGRP) activation on BNST activity is absent, and the decrease post activation is potentiated. Thereby, our data suggest neuroadaptations in PBN (CGRP) → BNST are sensitive to a previous stress-induced anxiogenic state. This is highly relevant, as this is the first study to our knowledge to demonstrate neuroadaptations in PBN (CGRP) → EA circuit in the absence of peripheral noxious stimuli. Together with previous data, these findings inform the mechanistic role of PBN (CGRP) → BNST and provide evidence that the circuit is sensitive to neuroadaptations associated with a previous anxiogenic state. Given CGRP is a therapeutic target for neurobiological disorders (Edvinsson et al. 2018; Labastida-Ramirez et al. 2023; Sarkar et al. 2025) and a CGRP intervention before stress has long-lasting effects on affect (Hashikawa-Hobara et al. 2015), future studies will investigate its potential role in alleviating heightened anxiety.

## Data Availability

The data that support the findings of this study are available from the corresponding author upon reasonable request.

## Abbreviations:

aCSF: artificial cerebrospinal fluid
ANOVA: analysis of variance
AUC: area under the curve
BNST: bed nucleus of the stria terminalis
CeA: central amygdala
cFOS: protein c-Fos
CGRP: calcitonin gene-related peptide
CIE: chronic intermittent ethanol
CNMF-E: constrained nonnegative matrix factorization for microendoscopic data
CNO: clozapine-n-oxide
CRE: cyclic recombinase
CRF: corticotropin-releasing factor
EA: extended amygdala
EPSC: excitatory postsynaptic current
FSS: forced swim stress
GCaMP: green fluorescent protein–based calcium indicator
GFP: green fluorescent protein
hM3D Gq: human M3 muscarinic Gq-coupled
hM3D Gq DREADDs: human M3 muscarinic designer receptors exclusively activated by designer drugs
IP: intraperitoneal
IPSC: inhibitory postsynaptic current
NDS: normal donkey serum
NMDG: N-methyl-D-glucamine
NoRMCorre: nonrigid motion correction of calcium imaging data
NSFT: novelty-suppressed feeding test
PBN: parabrachial nucleus
PBS: phosphate buffered saline
PBST: 0.2% Triton X-100 in PBS
RM-ANOVA: repeated measures analysis of variance
RT: room temperature
SUD: substance use disorder
